# Immune Pathogenesis of Asymptomatic *Chlamydia trachomatis* Infections in the Female Genital Tract

**DOI:** 10.1155/S1064744995000548

**Published:** 1995

**Authors:** Steven S. Witkin

**Affiliations:** Division of Immunology and Infectious Diseases Department of Obstetrics and Gynecology Cornell University Medical College 515 East 71 Street New York 10021 USA

## Abstract

*Chlamydia trachomatis* (CT) infections of the female genital tract, although frequently asymptomatic, are a major cause of fallopian-tube occlusion and infertility. Early stage pregnancy loss may also be due to an unsuspected and undetected CT infection. In vitro and in vivo studies have demonstrated that this organism can persist in the female genital tract in a form undetectable by culture. The mechanism of tubal damage as well as the rejection of an embryo may involve an initial immune sensitization to the CT 60 kD heat shock protein (HSP), followed by a reactivation of HSP-sensitized lymphocytes in response to the human HSP and the subsequent release of inflammatory cytokines. The periodic induction of human HSP expression by various microorganisms or by noninfectious mechanisms in the fallopian tubes of women sensitized to the CT HSP may eventually result in tubal scarring and occlusion. Similarly, an immune response to human HSP expression during the early stages of pregnancy may interfere with the immune regulatory mechanisms required for the maintenance of a semiallogeneic embryo.


Infectious Diseases in Obstetrics and Gynecology 3:169–174 (1995)
© 1995 Wiley-Liss, Inc.

### Immune Pathogenesis of Asymptomatic *Chlamydia trachomatis* Infections in the Female Genital Tract

Steven S. Witkin

*Division of Immunology and Infectious Diseases, Department of Obstetrics and Gynecology, Cornell University Medical College, New York, NY*

ABSTRACT

***Chlamydia trachomatis* (CT) infections of the female genital tract, although frequently asymptomatic, are a major cause of fallopian-tube occlusion and infertility. Early stage pregnancy loss may also be due to an unsuspected and undetected CT infection. In vitro and in vivo studies have demonstrated that this organism can persist in the female genital tract in a form undetectable by culture. The mechanism of tubal damage as well as the rejection of an embryo may involve an initial immune sensitization to the CT 60 kD heat shock protein (HSP), followed by a reactivation of HSP-sensitized lymphocytes in response to the human HSP and the subsequent release of inflammatory cytokines. The periodic induction of human HSP expression by various microorganisms or by noninfectious mechanisms in the fallopian tubes of women sensitized to the CT HSP may eventually result in tubal scarring and occlusion. Similarly, an immune response to human HSP expression during the early stages of pregnancy may interfere with the immune regulatory mechanisms required for the maintenance of a semiallogeneic embryo.** © 1995 Wiley-Liss, Inc.

KEY WORDS

Heat shock protein, spontaneous abortion, fallopian-tube occlusion, infertility

Most women who are infertile as a result of fallopian-tube occlusion have never been diagnosed as having sexually transmitted diseases. In a recent study of 283 women with tubal-factor infertility, 84% had no history consistent with an upper-genital-tract infection.[1] However, the degree of tubal damage in asymptomatic women was as severe as in women with symptomatic tubal infections. The involvement of *Chlamydia trachomatis* (CT) in tubal damage was indicated by the greatly increased prevalence of antibodies to this organism in women with asymptomatic tubal infertility.[2,3]

This article focuses on the experimental data consistent with the hypothesis that a long-term, undetected, asymptomatic CT infection of the female genital tract both induces tubal damage and interferes with the early stages of pregnancy through an autoimmune mechanism.

### PROBLEMS IN DETECTION OF ASYMPTOMATIC CT INFECTION

In addition to being asymptomatic, CT infections are difficult to detect. Culture, the traditional "gold standard" for CT detection, is a time-consuming, technically challenging procedure because of the obligate requirement of this organism for intracellular growth within a mammalian cell. The occasional presence of inhibitors of mammalian cell growth in the tissue culture of a genital-tract sample leads to false-negative results. Antigen detection assays or DNA hybridization reactions, although more rapid than culture, suffer from a decreased sensitivity.[4] A recently developed polymerase chain

**Address correspondence/reprint requests to Dr. Steven S. Witkin, Department of Obstetrics and Gynecology, Cornell University Medical College, 515 East 71 Street, New York, NY 10021.**

Received June 8, 1995

Accepted September 14, 1995

Review Article

TABLE 1. Properties of the CT 60 kD HSP

| |
|---|
| Second most abundant CT protein in cell lysates |
| Loosely associated with cell surface |
| Present in both reticulate and elementary bodies |
| Only protein synthesized in nonproductive latent infections induced by IFNγ |
| Member of highly conserved 60 kD family of HSP |
| 50% amino acid sequence homology to the human 60 kD HSP |
| Potent inducer of inflammation in animals previously infected with CT |

reaction (PCR) for CT (Amplicor, Roche Molecular Systems, Branchburg, NJ), approved by the Food and Drug Administration, has now replaced the culture as the "gold standard" for detection of this organism.[5]

Recent evidence has indicated that CT may reside in the fallopian tubes in a form that cannot be cultured. In vitro, the addition of interferon-gamma (IFN-γ) to CT-infected cells led to the formation of aberrant reticulate bodies that were unable to proliferate. These forms produced minimal amounts of CT structural antigens but continued to synthesize and release the 60 kD heat shock protein (HSP). Removal of the IFN-γ, however, led to their reversion to morphologically normal reticulate bodies which then differentiated into the infectious elementary body forms of the organism.[6] The detection of CT DNA in the inflamed fallopian tubes of women with tubal occlusions who were negative for CT by culture has also been reported.[7] Moreover, the findings of localized inflammation and CT DNA or protein in the absence of a positive CT culture have been reported for trachoma[8] and reactive arthritis.[9]

### CT 60 kD HSP

As mentioned above, the 60 kD HSP is the major CT protein produced during a chronic, nonproductive infection. The properties of this protein are listed in Table 1. Of major interest was the demonstration of this purified protein's ability to elicit a potent inflammatory response at sites distant from the original infection, in previously infected nonhuman primates,[10] indicating that a CT genital-tract infection could prime an individual to respond to the appearance of HSP in the absence of an active infection.

A second relevant property of the CT HSP is its similarity to human HSP in having an amino acid sequence of approximately 50% homology. It has an even stronger homology to the HSP of other pathogenic microorganisms.[11] Thus, an immune reaction to the CT HSP can sensitize lymphocytes to crossreacting epitopes in the HSP of man as well as other bacteria.

The human 60 kD HSP is normally sequestered within the mitochondria where it functions in protein transport and assembly and the regulation of ATPase activity.[12] However, under conditions of cellular stress due to increased temperature, infection, inflammation, or other conditions, the HSP is expressed elsewhere within the cell and on the cell's surface.[13] The HSP binds to other proteins and prevents their denaturation until the stressful stimuli are alleviated.

### IMMUNE RESPONSE TO THE CT HSP IN WOMEN WITH SALPINGITIS

Several groups of investigators have demonstrated that a humoral immune response to the CT HSP was associated with occlusion of the fallopian tubes.[14–16] We subsequently determined that lymphocytes from most women without evidence of CT exposure as well as from women with CT infections confined to the cervix did not respond to the CT HSP or to synthetic peptide epitopes of this protein. In contrast, the lymphocytes from a high percentage of women with recurrent episodes of salpingitis exhibited cell-mediated immune responses to conserved HSP epitopes.[17,18] This finding suggested that repeated or chronic exposure to the CT HSP was necessary to sensitize women to the epitopes also present in the human protein.

Once a woman becomes immunized to conserved epitopes of the CT HSP, any event that induces the expression of a crossreacting HSP will reactivate the sensitized lymphocytes. The observation that, in many cases of pelvic inflammatory disease, no infecting microorganism can be detected[19] may be partially explained by an autoimmune mechanism of inflammation involving prior sensitization to the CT HSP and the subsequent induction of human HSP expression in the fallopian tubes as a consequence possibly of fever (elevated temperature) or a localized immune-system activation of noninfectious origin. The ascension of microorganisms not typically associated with pelvic infections into the fallopian tubes may possibly induce human HSP expression by a similar mechanism. Repeated episodes of immune activation and inflammatory cytokine release would eventually damage the epithelium and induce scar formation and occlusion.

The ability of CT to exist intracellularly in a viable but nonproliferative state[6] may also contribute to chronic inflammatory-mediated damage. The immune response to a CT upper-genital-tract infection includes the release of IFN-$\gamma$[20] and tumor necrosis factor alpha (TNF$\alpha$).[21] The 60 kD HSP is also a strong inducer of inflammatory cytokines.[22] IFN-$\gamma$ will inhibit the continued development of CT reticulate bodies and lead to the formation of aberrant intracellular forms.[6] In addition, it will induce the expression of class-2 major histocompatibility complex (MHC) antigens on the surface of epithelial cells and macrophages. As suggested previously,[20] this reaction fosters the induction of inflammatory responses directed against epithelial membrane antigen-MHC complexes. The TNF$\alpha$ will interfere with fibroblast and epithelial-cell growth and further activate phagocytic cells to release products that are cytotoxic to epithelial cells. During this period of immune stimulation, the extracellular CT elementary bodies will be eradicated by the immune system. When this stage is completed, IFN-$\gamma$ and TNF$\alpha$ production will cease and the aberrant reticulate forms will resume normal development. A recommencement of elementary-body release from the infected cells will reactivate the immune response and the reappearance of both cytokines. Repetitive cycles of active infection followed by dormancy will gradually erode the integrity of the tubal epithelia. The consequent increasing degree of scar formation will eventually compromise the patency of the fallopian tubes.

### IMMUNE RESPONSE TO THE CT HSP IN EARLY STAGE PREGNANCY LOSS

Women with histories of pelvic inflammatory disease who become pregnant were shown to have a greatly increased incidence of spontaneous abortion compared with matched control women.[23] To ascertain the role, if any, of CT in early stage pregnancy loss more directly, we initiated studies of women undergoing in vitro fertilization (IVF) at the New York Hospital-Cornell Medical Center. The utilization of an IVF population allowed us to pinpoint accurately the time of conception and embryo transfer and to monitor closely the progression of the pregnancy.

Cervical samples were obtained at the time of oocyte retrieval from 307 IVF patients who were negative for CT by culture. These samples were reassayed by PCR for evidence of this organism.[24] Twenty (6.5%) of the women were positive for CT by PCR. The presence of CT was associated both with a failure to become pregnant after embryo transfer and with a spontaneous abortion after successful implantation. This study reinforced the clinical relevancy of the culture-negative, PCR-positive detection of CT and provided data that an asymptomatic CT infection may be a cause of early stage pregnancy loss.

To examine the possible role in pregnancy loss of an immune response to the CT HSP, we analyzed cervical samples from 216 IVF patients for cervical IgA antibodies to the recombinant CT HSP as well as to CT structural antigens.[25] Term deliveries occurred in 34.3% of the 198 patients who proceeded to embryo transfers; only 5 (7.3%) of these women were positive for anti-HSP IgA and 1 (1.5%) was positive for IgA to CT structural components. In contrast, the presence of cervical antibodies to both HSP and CT structural components strongly correlated with a poor IVF outcome. Of the women whose embryo transfers did not lead to term deliveries, anti-HSP IgA was present in 36 (27.7%) and anti-CT IgA in 24 (16.2%).

Almost all of the women sensitized to CT structural antigens also had antibodies to HSP. However, only 35% of the women with cervical IgA antibodies to HSP were sensitized to the other CT antigens. This finding suggested 3 possibilities: 1) that the HSP was the most immunogenic of the CT antigens, 2) that a latent CT infection was present in which only HSP was being produced,[6] or 3) that the women were sensitized to the HSP from an organism other than CT and crossreacting antibodies were being detected.

The demonstration that local cervical immunity to CT was associated with a poor IVF outcome confirmed our previous studies using PCR to detect this organism.[25] We believe it is unlikely that the presence of either CT or anti-CT antibodies in the cervix was responsible for the observed pregnancy failures. A more likely explanation is that their detection serves as a marker for the concomitant presence of this organism in the upper genital tract where infection or immune sensitization would be responsible for early stage pregnancy termination.

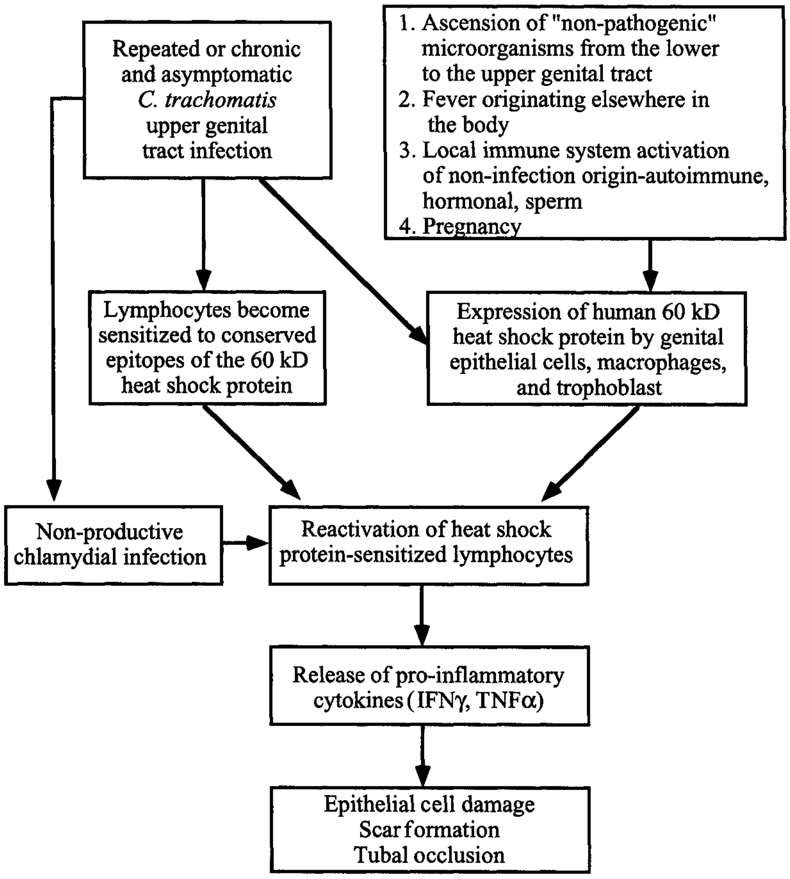

**Fig. 1. Proposed mechanism of CT-induced autoimmune-mediated fallopian-tube occlusion or early stage pregnancy loss.**

There is evidence that the human 60 kD HSP is expressed on the surface of decidual epithelial cells during early pregnancy.[26] Similarly, murine hybridomas specifically responsive to the 60 kD HSP have been shown to react with human trophoblasts.[27] Therefore, there appears to be ample human HSP expression during the early stages of pregnancy to activate HSP-sensitized lymphocytes and induce a localized, autoimmune inflammatory response.

The proposed sequence of events leading from a CT infection to immune sensitization to conserved HSP epitopes to tubal scarring or pregnancy failure is illustrated in Figure 1.

### CT INFECTION AND ANTISPERM ANTIBODIES

Women with circulating antibodies against their partners' motile sperm have a reduced probability for conception.[28] Fortunately, because of the immunosuppressive properties of sperm and seminal fluid,[29] most sexually active women do not produce antisperm antibodies. However, the wives of men with asymptomatic CT infections detected by PCR have been shown to have an increased prevalence of IgG and IgA antisperm antibodies in their sera.[30] Most probably, the CT, which can nonspecifically adhere to sperm,[31] acts as an adjuvant in inducing an immune response to both CT and sperm-specific antigens.[32] Therefore, another consequence of an asymptomatic genital-tract CT infection may be an increased susceptibility to the development of antisperm antibodies and immunological infertility.

When a woman who is sensitized to sperm, thus having antisperm antibodies, engages in coitus, the production of IFN-γ is stimulated[33] by the interaction between antibody-coated sperm and T lymphocytes.[34] Therefore, when sperm reach the fallopian tubes of such a woman, they can interact with resi-

**TABLE 2. Screening for CT in asymptomatic sexually active women**

| |
|---|
| Whenever there is a new sexual partner |
| When there is unexplained infertility or spontaneous abortion |
| When either partner of an infertile couple has antisperm antibodies |
| Prior to an IVF cycle |
| When a pregnant woman has a history of sexually transmitted disease or infertility |

dent lymphocytes,[35] producing a localized increase in the IFN-$\gamma$ concentration and, as mentioned above, the induction of inflammatory responses to epithelial-cell membrane components. The subsequent expression of HSP at the sites of inflammation would result in a further exacerbation of localized tissue damage in women sensitized to HSP.

### SCREENING FOR CT GENITAL-TRACT INFECTIONS

In light of the asymptomatic nature of most CT genital-tract infections as well as the ability of this organism to evade immunological defenses, persist for long periods,[36] and gradually ascend from the lower to the upper genital tract, the most prudent means to reduce the incidence of CT-related infertility and pregnancy loss is the periodic screening of asymptomatic women (and men) who are at risk for acquiring this infection. Through such screening, newly acquired infections of the cervix (and the male genital tract) could be identified and antibiotic treatment promptly initiated prior to the infection of other regions of the genital tract and resultant immune sequelae.

Various studies have been published predicting which groups of women would most benefit from selective CT screening or advocating universal screening.[37–41] Further verification among different subpopulations of women will help to pinpoint future testing priorities. In addition, a more concerted effort is needed to educate women about the prevalence of CT infection, the lack of symptoms following an infection, the serious consequences of harboring an undetected infection, and the indications for screening. Our suggestions for CT testing in asymptomatic women are listed in Table 2.

Only through the education of patients and health-care providers coupled with the identification and prompt, effective treatment of *asymptomatic* female and male carriers of CT genital-tract infections can we hope to diminish the incidence of CT-associated infertility and pregnancy complications.

### REFERENCES

1. Cates W Jr, Joesoef MR, Goldman B: Atypical pelvic inflammatory disease: Can we identify predictors? Am J Obstet Gynecol 169:341–346, 1993.
2. Jones RB, Ardery BR, Hui SL, et al.: Correlation between serum antichlamydial antibodies and tubal factor as a cause of infertility. Fertil Steril 38:553–558, 1982.
3. Gump DW, Gibson M, Ashikaga T: Evidence of prior pelvic inflammatory disease and its relationship to *Chlamydia trachomatis* antibody and intrauterine contraceptive device use in infertile women. Am J Obstet Gynecol 146:153–158, 1983.
4. Ossewaarde JM, Rieffe M, Rozenberg-Arska M, Ossenkoppele PM, Nawrocki RP, Van Loon AM: Development and clinical evaluation of a polymerase chain reaction test for detection of *Chlamydia trachomatis*. J Clin Microbiol 30:2122–2128, 1992.
5. Mahony JB, Luinstra KE, Sellors JW, et al.: Role of confirmatory PCRs in determining performance of *Chlamydia* Amplicor PCR with endocervical specimens from women with a low prevalence of infection. J Clin Microbiol 32:2490–2493, 1994.
6. Beatty WL, Byrne GI, Morrison RP: Morphologic and antigenic characterization of interferon $\gamma$-mediated persistent *Chlamydia trachomatis* infection in vitro. Proc Natl Acad Sci USA 90:3998–4002, 1993.
7. Patton DL, Askienazy-Elbhar M, Henry-Suchet J, et al.: Detection of *Chlamydia trachomatis* in fallopian tube tissue in women with postinfectious tubal infertility. Am J Obstet Gynecol 171:95–101, 1994.
8. Holland SM, Hudson AP, Bobo L, et al.: Demonstration of chlamydial RNA and DNA during a culture-negative state. Infect Immun 60:2040–2047, 1992.
9. Keat A, Dixey J, Soonex C, Thomas B, Osborn M, Taylor-Robinson D: *Chlamydia trachomatis* and reactive arthritis: The missing link. Lancet 1:72–74, 1987.
10. Patton DL, Sweeney YT, Kuo CC: Demonstration of delayed hypersensitivity in *Chlamydia trachomatis* salpingitis in monkeys: A pathogenic mechanism of tubal damage. J Infect Dis 169:680–683, 1994.
11. Morrison RP, Manning DS, Caldwell HD: Immunology of *Chlamydia trachomatis* infections. Immunoprotective and immunopathogenetic responses. In Quinn TC (ed): Sexually Transmitted Diseases. New York: Raven Press, pp 57–84, 1992.
12. Luis AM, Alconada A, Cuezva JM: The $\alpha$ regulatory subunit of the mitochondrial F1-ATPase complex is a heat-shock protein. J Biol Chem 265:7713–7715, 1990.
13. Kiessling R, Gronberg A, Ivanyi J, et al.: Role of hsp60 during autoimmune and bacterial inflammation. Immunol Rev 121:91–111, 1991.
14. Brunham RC, Maclean IW, Binns B, Peeling RW: *Chlamydia trachomatis:* Its role in tubal infertility. J Infect Dis 152:1275–1282, 1985.

15. Wager EA, Schachter J, Bavoil P, Stephens RS: Differential human serologic response to two 60,000 molecular weight *Chlamydia trachomatis* antigens. J Infect Dis 162: 922–927, 1990.
16. Toye B, Laferriere C, Claman P, Jessamine P, Peeling R: Association between antibody to the chlamydial heat-shock protein and tubal infertility. J Infect Dis 168:1236–1240, 1993.
17. Witkin SS, Jeremias J, Toth M, Ledger WJ: Cell-mediated immune response to the recombinant 57-kDa heat-shock protein of *Chlamydia trachomatis* in women with salpingitis. J Infect Dis 167:1379–1383, 1993.
18. Witkin SS, Jeremias J, Toth M, Ledger WJ: Proliferative response to conserved epitopes of the *Chlamydia trachomatis* and human 60-kilodalton heat shock proteins by lymphocytes from women with salpingitis. Am J Obstet Gynecol 171:455–460, 1994.
19. Expert Committee on Pelvic Inflammatory Disease: Pelvic inflammatory disease. Research directions for the 1990s. Sex Transm Dis 18:46–64, 1991.
20. Grifo JM, Jeremias J, Ledger WJ, Witkin SS: Interferon-γ in the diagnosis and pathogenesis of pelvic inflammatory disease. Am J Obstet Gynecol 160:26–31, 1989.
21. Toth M, Jeremias J, Ledger WJ, Witkin SS: In vivo tumor necrosis factor production in women with salpingitis. Surg Gynecol Obstet 174:359–362, 1992.
22. Retzlaff C, Yamamoto Y, Hoffman PS, Friedman H, Klein TW: Bacterial heat shock proteins directly induce cytokine mRNA and interleukin-1 secretion in macrophage cultures. Infect Immun 62:5689–5693, 1994.
23. Toth M, Chaudhry A, Ledger WJ, Witkin SS: Pregnancy outcome following pelvic infection. Infect Dis Obstet Gynecol 1:12–15, 1993.
24. Witkin SS, Kligman I, Grifo JM, Rosenwaks Z: *Chlamydia trachomatis* detected by polymerase chain reaction in cervices of culture-negative women correlates with adverse in vitro fertilization outcome. J Infect Dis 171: 1657–1659, 1995.
25. Witkin SS, Sultan KM, Neal GS, Jeremias J, Grifo JA, Rosenwaks Z: Unsuspected *Chlamydia trachomatis* infection and in vitro fertilization outcome. Am J Obstet Gynecol 171:1208–1214, 1994.
26. Mincheva-Nilsson L, Baranov V, Yeung MM, Hammarstrom S, Hammarstrom ML: Immunomorphologic studies of human decidua-associated lymphoid cells in normal early pregnancy. J Immunol 152:2020–2032, 1994.
27. Heyborne K, Fu YX, Nelson A, Farr A, O'Brien R, Born W: Recognition of trophoblasts by γδ T cells. J Immunol 153:2918–2926, 1994.
28. Witkin SS, David SS: Effect of antisperm antibodies on pregnancy outcome in a subfertile population. Am J Obstet Gynecol 158:59–62, 1988.
29. Witkin SS: Mechanisms of active suppression of the immune response to spermatozoa. Am J Reprod Immunol Microbiol 17:61–64, 1988.
30. Witkin SS, Jeremias J, Grifo JA, Ledger WA: Detection of *Chlamydia trachomatis* in semen by the polymerase chain reaction in male members of infertile couples. Am J Obstet Gynecol 168:1457–1462, 1993.
31. Wolner-Hanssen P, Mardh PA: In vitro test of adherence of *Chlamydia trachomatis* to human spermatozoa. Fertil Steril 42:102–107, 1984.
32. Munoz MG, Witkin SS: Autoimmunity to spermatozoa, asymptomatic *Chlamydia trachomatis* genital tract infection and γδ T lymphocytes in seminal fluid from the male partners of couples with unexplained infertility. Hum Reprod 10:1070–1074, 1995.
33. Witkin SS, Chaudhry A: Circulating interferon-γ in women sensitized to sperm: New mechanisms of infertility. Fertil Steril 52:867–869, 1989.
34. Witkin SS: Production of interferon gamma by lymphocytes exposed to antibody-coated spermatozoa: A mechanism for sperm antibody production in females. Fertil Steril 50:498–502, 1988.
35. Boehme M, Donat H: Identification of lymphocyte subsets in the human fallopian tube. Am J Reprod Immunol 28:81–84, 1992.
36. McCormack WM, Alpert S, McComb DE, Nichols RL, Semine DZ, Zinner SH: Fifteen-month follow-up study of women infected with *Chlamydia trachomatis*. N Engl J Med 300:123–125, 1979.
37. Handsfield HH, Jasman LL, Roberts PL, Hanson VW, Kothenbeutel RL, Stamm WE: Criteria for selective screening for *Chlamydia trachomatis* infection in women attending family planning clinics. JAMA 255:1730–1734, 1986.
38. Rahm VA, Odlind V, Pettersson R: *Chlamydia trachomatis* in sexually active teenage girls. Factors related to genital chlamydia infection: A prospective study. Genitourin Med 67:317–321, 1991.
39. Weinstock HS, Bolan GA, Kohn R, Balladares C, Back A, Oliva G: *Chlamydia trachomatis* infection in women: A need for universal screening in high prevalence populations? Am J Epidemiol 135:41–47, 1992.
40. Stergachis A, Scholes D, Heidrich FE, Sherer DM, Holmes KK, Stamm WE: Selective screening for *Chlamydia trachomatis* infection in a primary care population of women. Am J Epidemiol 138:143–153, 1993.
41. Hillis SD, Nakashima A, Marchbanks PA, Addiss DG, Davis JP: Risk factors for recurrent *Chlamydia trachomatis* infections in women. Am J Obstet Gynecol 170:801–806, 1994.
